# Validation of a Neurophysiological-Based Wearable Device (Somfit) for the Assessment of Sleep in Athletes

**DOI:** 10.3390/s25072123

**Published:** 2025-03-27

**Authors:** Gregory D. Roach, Dean J. Miller, Stephanie J. Shell, Kathleen H. Miles, Charli Sargent

**Affiliations:** 1 Appleton Institute for Behavioural Science, Central Queensland University, Adelaide 4701, Australia; 2 Australian Institute of Sport, Australian Sports Commission, Canberra 2617, Australia; 3 School of Medicine and Psychology, The Australian National University, Canberra 2601, Australia

**Keywords:** athlete, sleep, wearable, polysomnography, validation, agreement, sensitivity, specificity, Bland–Altman, error matrix

## Abstract

The aim of the study was to examine the validity of a neurophysiological-based wearable device, i.e., Somfit (Compumedics Ltd.), for the assessment of sleep in athletes. Twenty-seven athletes (14 F, 13 M, aged 22.3 ± 5.1 years) spent a single night in a sleep laboratory. The participants had 9 h in bed (23:00–08:00) while fitted simultaneously with Somfit and polysomnography (PSG), i.e., the gold standard for the assessment of sleep. Somfit and PSG were used to independently categorise each 30-s epoch of time in bed into one of five states, i.e., wake, stage 1 non-REM sleep (N1), stage 2 non-REM sleep (N2), stage 3 non-REM sleep (N3), or REM sleep. There were large differences between participants in terms of the amount of Somfit data that were successfully captured/scored, so three subsets were considered in the subsequent analyses: unfiltered subset (n = 26)—all participants, except one for whom no Somfit data were captured/scored; good-capture subset (n = 15)—participants for whom > 80% of Somfit data were captured/scored; excellent-capture subset (n = 7)—participants for whom > 99.9% of Somfit data were captured/scored. Agreement for the five-state categorisation of time in bed was calculated as the percentage of PSG epochs correctly scored by Somfit as N1, N2, N3, REM, or wake. Agreement (and Cohen’s kappa) was 63% (0.47) for the unfiltered subset, 66% (0.52) for the good-capture subset, and 79% (0.70) for the excellent-capture subset. These data indicate a moderate–substantial level of agreement between Somfit and PSG for the assessment of sleep in athletes. Wearable devices that can capture valid sleep data may also be used to derive important measures related to the circadian system, such as sleep consistency and social jet lag.

## 1. Introduction

Polysomnography (PSG) is the gold standard technique for the assessment of human sleep [1]. At a minimum, PSG requires electrodes to be attached to the scalp, face, and body to generate signals for electroencephalogram (EEG), electrooculogram (EOG), and electromyogram (EMG). A trained sleep technologist then manually scores each 30 s segment (epoch) of a sleep period as either wake, light sleep (stage 1 non-REM sleep [N1] or stage 2 non-REM sleep [N2]), deep sleep (stage 3 non-REM sleep [N3]), or rapid eye movement (REM) sleep based on characteristic patterns of brain activity in the EEG, eye movements in the EEG, and muscle tone in the EMG. Measures regarding the quantity and quality of sleep can then be derived from the scored PSG records (total sleep time, amount of deep sleep, etc.).

Various wearable devices, i.e., sleep wearables, have been developed as alternatives to PSG for the assessment of sleep because the collection of PSG data can be complex, inconvenient, time-consuming, and costly. There are two main types of sleep wearables. Activity-based wearables are worn on the body, typically on the wrist or finger, and base sleep assessments on measures of physical activity alone (in the case of research-grade wearables), or a combination of physical activity and cardiac activity (in the case of commercial-grade wearables). Neurophysiological-based wearables are worn on the head, typically on the forehead, and base sleep assessments on measures of brain activity, eye movements and muscle tone—similar to the measures used in PSG, but using a smaller number of electrodes that can be self-applied.

In preparation for a previous validation study [2], high-performance sport staff identified the top six sleep wearables that were being used or were likely to be used in the near future by elite athletes. The devices that were identified included five commercial-grade activity-based wearables, from Apple, Garmin, Polar, Oura and Whoop, and one neurophysiological-based wearable, from Compumedics. The validity of sleep wearables from these six providers was examined in numerous separate studies with non-athlete participants. The results of these studies indicate that the neurophysiological-based wearable, i.e., Somfit by Compumedics, has a higher level of agreement with PSG than the activity-based wearables, e.g., [3,4,5,6]. Similarly, when devices from all six providers were worn simultaneously by fifty physically active participants during a single night of sleep, Somfit had a higher level of agreement with PSG than the activity-based wearables [2]. These outcomes are probably to be expected as the measures used by Somfit to assess sleep are similar to those used by PSG.

There is some evidence to indicate that athletes have poorer sleep quality than non-athletes [7,8], and it is well-established that sleep wearables are less accurate when used in populations with poor-quality sleep compared to others [9,10,11]. Therefore, when assessing the validity of sleep wearables that are likely to be used by athletes, it is important to include them as participants in the research. A limited number of studies have assessed the validity of activity-based wearables to assess sleep with athletes wearing research-grade devices [12,13] and commercial-grade devices [14,15], but the validity of neurophysiological-based sleep wearables has not been tested specifically in athletes. In response to this gap, the objective of the current study was to examine the validity of a neurophysiological-based wearable, i.e., Somfit, for assessing athletes’ sleep by comparing its outputs with PSG.

## 2. Materials and Methods

### 2.1. Participants

Twenty-seven participants completed the study [14 female; 13 male; age = 22.3 ± 5.1 years (mean ± SD)]. To be included, participants had to be (a) well-trained athletes (n = 4), i.e., participating in at least 2 h of training per day, at least 3 days per week, for at least 3 years [16], or (b) highly trained athletes (n = 23), i.e., participating in at least 3 h of training per day, at least 5 days per week, for at least 5 years [16]. Participants were recruited from the sports of soccer (n = 10), beach volleyball (n = 6), athletics (n = 3), basketball (n = 3), diving (n = 2), orienteering (n = 1), track cycling (n = 1), and weightlifting (n = 1). Wearable devices may be used to assess athletes’ sleep at any phase of the yearly competition cycle, so the sample in this study included participants in all three phases of the cycle, i.e., pre-season (n = 10), in-season (n = 14), and off-season (n = 3). Participants provided written informed consent and received a nominal honorarium for their involvement (in the form of a gift voucher). This study was approved by the CQUniversity Human Research Ethics Committee following the guidelines of the National Health and Medical Research Council (Australia).

### 2.2. Equipment

#### 2.2.1. Polysomnography (Gold Standard)

The gold standard assessment of sleep was conducted using PSG. In this study, a standard montage of Grass gold-cup electrodes (AstroMed, West Warwick, RI, USA) was attached to each participant’s scalp, face, and body. The montage included four channels of electroencephalography (EEG) to assess brain activity (C4–M1, C3–M2, F4–M1, O2–M1), two electrooculograms (EOG) to assess eye movements (left/right outer canthus), and three electromyograms (EMG) to assess muscle tone (submental). PSG data were transmitted via ethernet and recorded to a Grael data acquisition, storage, and analysis system (Compumedics Ltd., Melbourne, Australia).

#### 2.2.2. Somfit (Wearable Device)

Somfit (Compumedics Ltd., Melbourne, Australia) is a wearable device that attaches to the forehead through a self-adhesive, disposable patch. The device records various data, including two EEG signals, pulse rate, oxygen saturation, motion, ambient light, skin temperature, ambient temperature, etc. The data are transmitted via Bluetooth to a mobile phone paired to the Somfit device and then transmitted via Wi-Fi to a cloud-based data management and reporting system (Profusion Nexus360, version 2.0.0.34).

### 2.3. Procedure

The study was conducted at The Sleep Lab at CQUniversity’s Appleton Institute for Behavioural Science in Wayville, South Australia. The Sleep Lab comprises two accommodation suites fitted out as serviced apartments. In total, the suites contain six bedrooms, six bathrooms, two kitchens, two dining rooms, a gymnasium, and laundry facilities. The accommodation suites are sound-attenuated, windowless, and temperature-controlled, such that, during time in bed, background noise was 30 dB, bedrooms were completely dark, and the target ambient temperature was 21–23 °C.

Each participant attended The Sleep Lab on a single night in groups of 4–6 people. Participants arrived after dinner in the evening (~20:00), were fitted with the PSG and Somfit equipment from 21:30 to 22:30, were given a 9 h sleep opportunity in bed with lights out from 23:00 to 08:00, and departed at ~08:30 after the sleep monitoring equipment was removed. To ensure the timing of the PSG and Somfit equipment was synchronised, all devices were set to the same time prior to each night of use.

### 2.4. Measures

#### 2.4.1. Epoch Scoring—Polysomnography (Gold Standard)

PSG records, from lights off to lights on, were independently scored by three trained, experienced sleep technologists. The records were scored according to version 2.6 of the AASM Recommended Rules [17] using Profusion PSG 3 (version 3.4.404) or Profusion PSG 5 (version 5.1.656) software (Compumedics Ltd., Melbourne, Australia). A five-state hypnogram was generated for each 9 h PSG record by each scorer, with all 1080 × 30-s epochs classified as one of five states (i.e., wake, N1, N2, N3, REM). The five-state hypnograms from the three scorers were used to create a five-state concordance hypnogram. If two or three of the scorers assigned the same sleep stage to a particular 30-s epoch, the corresponding epoch in the five-state concordance hypnogram was classified as that stage. If the three scorers assigned three different sleep stages to a particular 30-s epoch, the corresponding epoch in the five-state concordance hypnogram was classified as ‘discordant’. The five-state hypnograms from the three scorers were also converted into two-state hypnograms, with all 1080 × 30-s epochs classified as one of two states (i.e., wake or sleep). The two-state hypnograms from the three scorers were used to create a two-state concordance hypnogram (employing a similar process as for the five-state concordance hypnogram).

#### 2.4.2. Epoch Scoring—Somfit (Wearable Device)

Somfit records, from lights off to lights on, were automatically scored independently of the PSG records using the Profusion Nexus360 cloud-based data management and reporting system. A five-state hypnogram was generated for each 9 h Somfit record, with all 1080 × 30-s epochs classified as one of five states (i.e., wake, N1, N2, N3, or REM). The five-state hypnograms were also converted into two-state hypnograms, with all 1080 × 30-s epochs classified as one of two states (i.e., wake or sleep).

#### 2.4.3. Summary Sleep Variables—Polysomnography and Somfit

For each participant’s night of sleep, the PSG five-state concordance hypnogram and the Somfit five-state hypnogram were used to determine two separate sets of variables regarding the amount of time spent in any stage of sleep (total sleep time) and the amount of wake, N1, N2, N3, and REM for each 9 h period in bed. N1 and N2 are lighter stages of sleep, and N3—sometimes referred to as slow-wave sleep—is a deeper stage of sleep [1]. REM sleep is sometimes referred to as dreaming sleep, but dreaming also occurs in non-REM sleep, albeit about half as often compared to REM sleep [18].

### 2.5. Data Analysis

An analysis of the data was conducted using Microsoft Excel for Mac version 16.74 and IBM SPSS Statistics version 27.0.1.0. The analysis was consistent with a standardised procedure for assessing the performance of sleep wearables [19].

#### 2.5.1. Epoch-by-Epoch Comparisons

Epoch-by-epoch comparisons of Somfit with PSG were performed for two-state categorisation of time in bed (as sleep or wake) and five-state categorisation of time in bed (as a particular sleep stage or wake). For both sets of comparisons, a 30-s epoch of time in bed was excluded from the analyses if (a) the PSG epoch was classified as ‘discordant’ in the respective concordance hypnogram (i.e., no agreement between the scorers), or (b) the Somfit epoch was not captured/scored.

For the two-state categorisation of sleep, each 30-s epoch was classified as one of four types based on the agreement (or not) of Somfit with PSG (Table 1), and the following variables were calculated:

- Sensitivity for sleep (%) = TS/(TS + FW) × 100 (i.e., the percentage of PSG sleep epochs correctly scored as sleep by Somfit).
- Sensitivity for wake (%) = TW/(TW + FS) × 100 (i.e., the percentage of PSG wake epochs correctly scored as wake by Somfit [sometimes referred to as specificity]).
- Agreement (%) = (TS + TW)/(TS + TW + FS + FW) × 100 (i.e., the percentage of all PSG epochs correctly scored as sleep or wake by Somfit).

For the five-state categorisation of sleep, each 30-s epoch was classified as one of twenty-five types based on the agreement (or not) of Somfit with PSG (Table 2), and the following variables were calculated:

- Sensitivity for N1 (%) = TN1/(TN1 + FW_N1_ + FN2_N1_ + FN3_N1_+ FR_N1_) × 100
  (i.e., the percentage of PSG N1 epochs correctly scored as N1 by Somfit).

- Sensitivity for N2 (%) = TN2/(TN2 + FW_N2_+ FN1_N2_+ FN3_N2_ + FR_N2_) × 100
  (i.e., the percentage of PSG N2 epochs correctly scored as N2 by Somfit).

- Sensitivity for N3 (%) = TN3/(TN3 + FW_N3_ + FN1_N3_ + FN2_N3_ + FR_N3_) × 100
  (i.e., the percentage of PSG N3 epochs correctly scored as N3 by Somfit).

- Sensitivity for REM (%) = TR/(TR + FW_R_ + FN1_R_ + FN2_R_ + FN3_R_) × 100
  (i.e., the percentage of PSG REM epochs correctly scored as REM by Somfit).

- Sensitivity for wake (%) = TW/(TW + FN1_w_ + FN2_w_ + FN3_w_ + FR_w_) × 100
  (i.e., the percentage of PSG wake epochs correctly scored as wake by Somfit [sometimes referred to as specificity]).

- Agreement (%) = (TW + TN1 + TN2 + TN3 + TR)/(TW + TN1 + TN2 + TN3 + TR + FW_N1_ + FW_N2_ + FW_N3_ + FW_R_ + FN1_W_ + FN1_N2_ + FN1_N3_ + FN1_R_ + FN2_W_ + FN2_N1_ + FN2_N3_ + FN2_R_ + FN3_W_ + FN3_N1_ + FN3_N2_ + FN3_R_ + FR_W_ + FR_N1_ + FR_N2_ + FR_N3_) × 100
  (i.e., the percentage of all PSG epochs correctly scored as N1, N2, N3, REM or wake, by Somfit).

The agreement statistic was also calculated as a measure of inter-rater agreement for the five-state hypnograms produced by the three trained sleep technologists (A, B, C), i.e., A v. B agreement, A v. C agreement, and B v. C agreement.

Sensitivity and agreement indicate the likelihood that a PSG epoch will be correctly identified by Somfit. Cohen’s kappa (κ) was also calculated to evaluate the agreement values relative to that which could be expected due to chance [20]. The kappa statistic was interpreted using standard guidelines: 0 to 0.20 = slight agreement; >0.20 to 0.40 = fair agreement; >0.40 to 0.60 = moderate agreement; >0.60 to 0.80 = substantial agreement; >0.80 to <1.00 = almost perfect agreement; and 1.00 = perfect agreement.

Due to the vagaries associated with aligning the timing of different types of sleep-recording equipment, synchronisation may be imperfect, so there may be minor differences in clock time between some pairs of Somfit v. PSG sleep records. To minimise the effect of these differences, the five-state agreement was examined with offset adjustments of 1–10 × 30-s epochs in both directions at the individual level and the offset with the highest agreement value was applied. For the 26 Somfit records, one required no offset, and 25 required an offset; the maximum offset applied was 10 × 30-s epochs; the average absolute offset for the 25 affected records was 4.9 × 30-s epochs; and the mean multi-state agreement for all 26 records was 3.6% higher when offsets were applied.

#### 2.5.2. Summary Sleep Variables (Bland–Altman Analyses, Means Comparisons)

Agreement between Somfit and PSG for total sleep time was examined using the limits of the agreement method [21]. Modified Bland–Altman plots were produced to display (a) the pairwise differences between Somfit- and PSG-derived values for total sleep time, (b) the mean difference between the Somfit- and PSG-derived values for total sleep time (bias), and (c) the 95% limits of agreement, i.e., bias ± (1.96 × SD). The plots were examined for proportional bias and heteroscedasticity using ordinary least squares regression and the Breusch–Pagan test, respectively. In cases where heteroscedasticity or proportional bias were present, this was noted but the bias and 95% limits of agreement were not adjusted in the plots.

For all summary sleep variables, Somfit-derived values were compared with PSG-derived values using mean differences (bias), mean absolute differences (absolute bias), paired-sample *t*-tests and effect sizes (based on Cohen’s d). For each variable, the Somfit means were considered to differ from the PSG means if *p* < .05 for the *t*-test and/or the 95% confidence interval for Cohen’s d did not include zero. Furthermore, for total sleep time, wearable-based estimates are typically considered to be clinically satisfactory if the absolute bias is <30 min [22].

## 3. Results

### 3.1. Data Capture and Data Quality

Twenty-seven participants completed the study. All data from one participant were excluded because no Somfit data were recorded. It could not be determined whether this instance of complete data loss was due to human error (by researchers or the participant) or failure of Somfit hardware, software, firmware, or systems.

For the 26 participants for whom Somfit data were available, there were large differences between participants in (a) the number of 30-s epochs for which data were captured in the Somfit records and (b) the number of captured 30-s epochs for which data were of sufficient quality to be scored (as wake or a particular stage of sleep) by Somfit’s auto-scoring algorithms. Preliminary analyses indicated these factors were likely to affect the quality of the Somfit outputs, so for all subsequent analyses, three subsets of data were created:

- Unfiltered subset (n = 26): contains Somfit records (and their matching PSG records) from all participants.
- Good-capture subset (n = 15): contains Somfit records (and their matching PSG records) from participants for whom > 80% of the 30-s epochs were captured/scored by Compumedics’ Profusion Nexus360 system.
- Excellent-capture subset (n = 7): contains Somfit records (and their matching PSG records) from participants for whom > 99.9% of the 30-s epochs were captured/scored by Compumedics’ Profusion Nexus360 system.

### 3.2. Epoch-by-Epoch Comparisons

For the two-state categorisation of sleep/wake, the percent agreement and Cohen’s kappa were 84% and 0.45 for the unfiltered subset, 89% and 0.51 for the good-capture subset, and 94% and 0.64 for the excellent-capture subset (Table 3). In comparison, agreement for the two-state categorisation between pairs of sleep technologists scoring the PSG records was 93–96% for the unfiltered subset, 94–97% for the good-capture subset, and 94–97% for the excellent-capture subset (Table 3).

For the five-state categorisation of sleep/wake, percent agreement and Cohen’s kappa were 63% and 0.47 for the unfiltered subset, 66% and 0.52 for the good-capture subset, and 79% and 0.70 for the excellent-capture subset (Table 3). In comparison, agreement for five-state categorisation between pairs of sleep technologists scoring the PSG records was 77–87% for the unfiltered subset, 78–87% for the good-capture subset, and 78–87% for the excellent-capture subset (Table 3).

Error matrices for the five-state categorisation indicate that wake, N1, and REM were the most difficult states for Somfit to identify (Table 4, Table 5 and Table 6). When Somfit worked most effectively (see the excellent-capture subset, Table 6), its main sources of error were classifying wake as REM, classifying N1 as N2 or wake, classifying N2 as N3, classifying N3 as N2, and classifying REM as N2.

### 3.3. Summary Variables (Bland–Altman Analyses, Bias, Absolute Bias)

**Figure 1:**
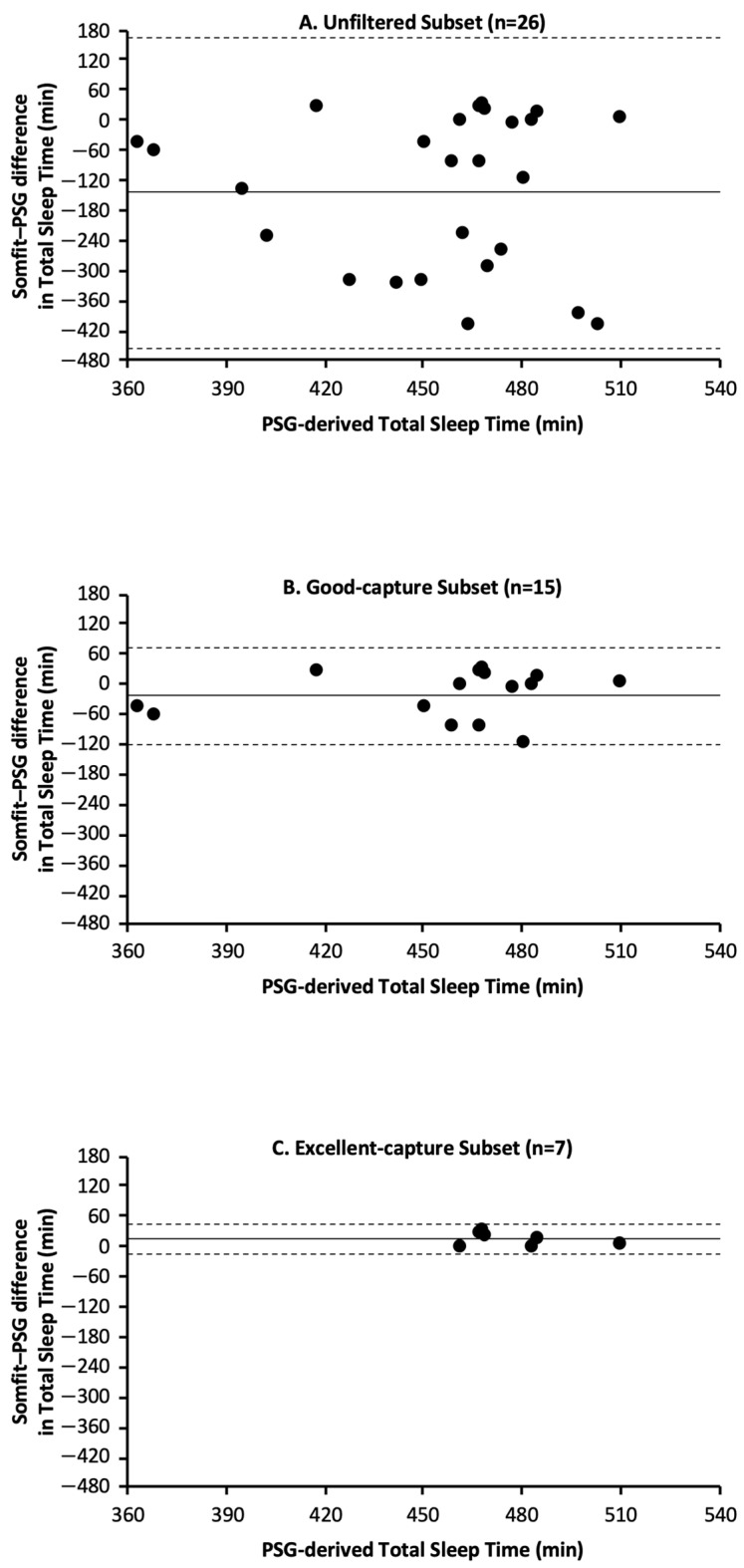
Bland–Altman plots for Somfit- and PSG-derived measures of TST based on data from the following: (**A**) unfiltered subset, (**B**) good-capture subset, and (**C**) excellent-capture subset. In each panel, positive values on the y axis indicate that Somfit overestimated TST relative to PSG and negative values on the y axis indicate that Somfit underestimated TST relative to PSG; the solid horizontal line indicates the Somfit mean bias relative to PSG and the horizontal dashed lines indicate the 95% limits of agreement (i.e., mean bias ± 1.96 standard deviations).

For the unfiltered subset, Somfit underestimated total sleep time (TST) by 144 min with an absolute bias of 152 min (Table 7, Figure 1A). For the good-capture subset, Somfit underestimated TST by 25 min with an absolute bias of 40 min (Table 8, Figure 1B). For the excellent-capture subset, Somfit overestimated TST by 10 min with an absolute bias of 14 min (Table 9, Figure 1C).

The modified Bland–Altman plots (Figure 1A–C) are presented in the standard fashion, with horizontal lines for mean bias and 95% confidence intervals. Regression analyses indicated that the three subsets did not have proportional bias, i.e., unfiltered subset—R^2^ = .006, df = 24, *p* = .70; good-capture subset—R^2^ = .046, df = 13, *p* = .44; and excellent-capture subset—R^2^ = .130, df = 5, *p* = .43. Breusch–Pagan tests indicated the unfiltered subset had heteroscedasticity, but the good-capture and excellent-capture subsets did not have heteroscedasticity, i.e., unfiltered subset—BP = 5.1, df = 1, *p* = .02; good-capture subset—BP = 0.6, df = 1, *p* = .45; and excellent-capture subset—BP = 2.6, df = 1, *p* = .10.

## 4. Discussion

### 4.1. Comparison of Somfit for Assessing the Sleep of Athletes and Non-Athletes

The results obtained in this validation study of Somfit with athletes are similar to those obtained in a previous validation study with non-athletes [2]. In the previous study, records were only included if >80% of the 30-s epochs had scoreable Somfit data—equivalent to the threshold used to determine the good-capture subset in the current study. In that study, Somfit correctly identified 65% of all PSG epochs for the five-state categorisation of sleep/wake, with a kappa value of 0.52, which indicates a moderate level of agreement. In comparison, for the Good-capture subset in the current study, Somfit correctly identified 66% of all PSG epochs, with a kappa value of 0.52. Together, these two sets of results indicate that Somfit is as good at estimating sleep staging during a full night of sleep for athletes as it is for non-athletes.

### 4.2. Validity of Somfit for Assessing Athletes’ Sleep

The degree to which Somfit can be considered valid for the assessment of sleep in athletes depends on which subset of the current data is given the greatest weighting. Consider the critical outcomes for each of the subsets of data. First, agreement between Somfit and PSG for the five-state categorisation of sleep/wake was 63% for the unfiltered subset, 66% for the good-capture subset, and 79% for the excellent-capture subset. Second, compared with PSG, Somfit had a mean bias in TST, such that it was underestimated by 144 min for the unfiltered subset and 25 min for the good-capture subset, and overestimated by 10 min for the excellent-capture subset. Third, compared with PSG, Somfit had an absolute bias in TST such that it differed by 152 min for the unfiltered subset, 40 min for the good-capture subset, and 14 min for the excellent-capture subset. Finally, compared with the PSG-derived values for the summary sleep variables, based on means comparisons and effect size analyses, the Somfit-derived values differed for 6 of the 6 variables for the unfiltered subset, 1 of the 6 variables for the good-capture subset, and 0 of the 6 variables for the excellent-capture subset. If Somfit performed at a similar level with athletes outside the current study as it did for the unfiltered subset, it would not be considered a valid measure of sleep. In contrast, if Somfit performed at a similar level with athletes outside the current study as it did for the excellent-capture subset, then it would be considered a valid measure of sleep. Indeed, the five-state agreement between Somfit and PSG for the excellent-capture subset (79%) was similar to the inter-rater agreement between one pair of the trained sleep technicians scoring the gold standard PSG (78%). In addition, for the excellent-capture subset, the absolute bias in the estimate of TST by Somfit in comparison to PSG (14 min) was well under the threshold of 30 min and considered clinically satisfactory [22]. Furthermore, in a recent laboratory-based validation, albeit with a clinical sample rather than a sample of athletes, the five-state agreement between Somfit and PSG was 76% [5], which indicates that Somfit can perform at a similar level in other settings as it did for the excellent-capture subset in the current study.

### 4.3. Maximise the Likelihood of Capturing Excellent Somfit Data

If Somfit is used to assess sleep in athletes (and others), efforts should be made to ensure the quantity and quality of the data captured more closely resemble this study’s excellent-capture subset, rather than the unfiltered subset. Based on the authors’ experiences using Somfit in laboratory- and field-based settings, there are two major modifiable factors that influence the likelihood of maximising the quantity and quality of captured data. First, the quality of the signals received from the adhesive forehead patch that contains the Somfit electrodes seems to be highly dependent on the preparation of the forehead before the adhesive patch is applied. If users are given the instruction to “scrub the forehead” with an alcohol wipe prior to applying the adhesive patch, rather than to “clean the forehead” (as per the manufacturer’s user guides), the connection between the Somfit electrode and the forehead is superior, and a greater amount of high-quality data are likely to be collected. Therefore, it is recommended that users are advised to “scrub the forehead with an alcohol wipe as hard as possible without causing pain—then pause and repeat”. Scrubbing, rather than cleaning, more closely mimics the process that sleep technologists use when preparing skin prior to attaching PSG electrodes. Second, the quantity of the data captured by Somfit is affected by the degree to which the Bluetooth connection is maintained throughout a sleep period. Data are not captured during any time when the Somfit device adhered to the forehead is not connected to its paired mobile phone. To minimise loss of Bluetooth connection, and any associated loss of data, users should (a) position the mobile phone such that it has a direct line of sight with the Somfit device on the forehead, and (b) take the mobile phone with them if leaving the bedroom during a sleep period (e.g., for a bathroom visit).

### 4.4. Types of Use of Somfit to Assess Athletes’ Sleep

There are two main circumstances in which it may be useful to assess an athlete’s sleep. First, the daily monitoring of sleep/wake behaviour—this typically involves tracking basic constructs such as the timing of sleep, total sleep time and sleep quality for weeks, months, or years to monitor for potential changes caused by training load, competition, travel, illness, injury, etc. Second, a one-off examination of sleep structure—this is typically undertaken if an athlete is feeling fatigued for no apparent reason, having difficulty obtaining a reasonable amount of good-quality sleep, or waking up tired after a full night of sleep. Given the anecdotal reports from some users regarding the potential discomfort associated with wearing Somfit’s adhesive forehead patch, particularly if it is used for successive sleeps, it is possible that Somfit may not be tolerated by some athletes for the daily monitoring of sleep. However, in situations where it is necessary to obtain data regarding the structure of an athlete’s sleep (i.e., amount of wake, light sleep, deep sleep, and REM within a sleep period) over a limited number of nights, then Somfit could be used if PSG is impractical. If Somfit is used with athletes as an alternative to PSG, steps should be taken to maximise the quantity and quality of data that are captured (see Section 4.3). In future, if a forehead patch is developed that provides sufficient adhesion but has a lower likelihood of discomfort for the wearer, then Somfit may become a more viable option for the daily monitoring of athletes’ sleep.

### 4.5. Use of Somfit to Capture Metrics Related to the Circadian System

Any wearable device, including Somfit, that can capture valid sleep data may also be used to derive important measures regarding the strength of the circadian system, i.e., the internal body clock. For example, two such measures are sleep consistency, i.e., the day-to-day variability in the start/end times of sleep, and social jet lag, i.e., the difference in the timing of sleep on work days and free days. In non-athletes, low sleep consistency is associated with poor mental health [23], impaired cognitive function [24], and increased risk of mortality [25]; and high social jet lag is associated with depression [26], obesity [27], and poor academic performance [28]. In future, it may be possible to use data obtained from Somfit, or other sleep wearables, to examine the potential effects of circadian disruption on important outcomes for athletes, such as mental well-being, physical performance, and risk of illness or injury.

## Figures and Tables

**Table 1:** Sleep/wake agreement matrix for two-state categorisation of time in bed.

		Somfit	
		Wake	Sleep
**PSG**	**Wake**	True Wake (TW)	False Sleep (FS)
	**Sleep**	False Wake (FW)	True Sleep (TS)

**Table 2:** Sleep/wake agreement matrix for five-state categorisation of time in bed.

		Somfit				
		Wake	N1	N2	N3	REM
**PSG**	**Wake**	True Wake (TW)	False N1 (FN1_W_)	False N2 (FN2_W_)	False N3 (FN3_W_)	False REM (FR_W_)
	**N1**	False Wake (FW_N1_)	True N1 (TN1)	False N2 (FN2_N1_)	False N3 (FN3_N1_)	False REM (FR_N1_)
	**N2**	False Wake (FW_N2_)	False N1 (FN1_N2_)	True N2 (TN2)	False N3 (FN3_N2_)	False REM (FR_N2_)
	**N3**	False Wake (FW_N3_)	False N1 (FN1_N3_)	False N2 (FN2_N3_)	True N3 (TN3)	False REM (FR_N3_)
	**REM**	False Wake (FW_R_)	False N1 (FN1_R_)	False N2 (FN2_R_)	False N3 (FN3_R_)	True REM (TR)

**Table 3:** Epoch-by-epoch statistics for the two-state and five-state categorisation of time in bed (TIB).

Variable		Unfiltered Subset (n = 26)	Good-Capture Subset (n = 15)	Excellent-Capture Subset (n = 7)
**2-state Categorisation of TIB**				
Somfit v. PSG				
	Sleep Sensitivity (%)	88.7 ± 8.3	93.1 ± 5.0	97.0 ± 1.7
	Wake Sensitivity (%)	59.8 ± 21.3	58.3 ± 19.1	65.8 ± 14.1
	Agreement (%)	83.6 ± 11.1	88.7 ± 6.1	93.8 ± 2.2
	Cohen’s Kappa (κ)	0.45 ± 0.27	0.51 ± 0.23	0.64 ± 0.12
Scorer v. Scorer (PSG)				
	A v. B Agreement (%)	96.4 ± 2.6	96.7 ± 3.0	97.1 ± 3.5
	A v. C Agreement (%)	92.9 ± 3.6	93.7 ± 2.6	94.3 ± 2.5
	B v. C Agreement (%)	94.7 ± 3.5	95.6 ± 2.6	96.2 ± 1.7
**5-state Categorisation of TIB**				
Somfit v. PSG				
	N1 Sensitivity (%)	18.5 ± 9.2	18.9 ± 9.1	24.8 ± 8.8
	N2 Sensitivity (%)	69.1 ± 18.6	72.9 ± 18.7	84.4 ± 10.1
	N3 Sensitivity (%)	60.8 ± 33.3	67.6 ± 29.5	84.3 ± 12.5
	REM Sensitivity (%)	53.3 ± 37.7	63.7 ± 31.4	80.2 ± 28.5
	Wake Sensitivity (%)	59.8 ± 21.3	58.3 ± 19.1	65.8 ± 14.1
	Agreement (%)	62.6 ± 19.5	66.2 ± 19.5	79.4 ± 10.4
	Cohen’s Kappa (κ)	0.47 ± 0.27	0.52 ± 0.28	0.70 ± 0.16
Scorer v. Scorer (PSG)				
	A v. B Agreement (%)	86.8 ± 4.6	86.9 ± 5.3	87.0 ± 6.4
	A v. C Agreement (%)	77.4 ± 5.8	77.7 ± 5.2	78.0 ± 5.5
	B v. C Agreement (%)	81.2 ± 5.8	81.7 ± 4.8	82.1 ± 3.3

**Table 4:** Error matrix for the five-state categorisation of time in bed (unfiltered subset, n = 26).

		Somfit (Unfiltered Subset)					
		Wake	N1	N2	N3	REM	Sum
**PSG**	**Wake**	60	7	17	5	10	100
	**N1**	28	19	36	4	13	100
	**N2**	10	5	69	9	6	100
	**N3**	10	2	22	61	5	100
	**REM**	17	2	22	5	53	100

Notes: Shaded cells in the 5 × 5 matrix indicate the likelihood (%) that a PSG sleep state was correctly identified by Somfit. Non-shaded cells in the 5 × 5 matrix indicate the likelihood (%) of different types of misclassification errors by Somfit.

**Table 5:** Error matrix for the five-state categorisation of time in bed (good-capture subset, n = 15).

		Somfit (Good-Capture Subset)					
		Wake	N1	N2	N3	REM	Sum
**PSG**	**Wake**	58	7	20	3	12	100
	**N1**	23	19	41	4	13	100
	**N2**	5	4	73	11	8	100
	**N3**	5	3	22	68	3	100
	**REM**	8	2	23	4	63	100

Notes: Shaded cells in the 5 × 5 matrix indicate the likelihood (%) that a PSG sleep state was correctly identified by Somfit. Non-shaded cells in the 5 × 5 matrix indicate the likelihood (%) of different types of misclassification errors by Somfit.

**Table 6:** Error matrix for the five-state categorisation of time in bed (excellent-capture subset, n = 7).

		Somfit (Excellent-Capture Subset)					
		Wake	N1	N2	N3	REM	Sum
**PSG**	**Wake**	66	9	11	2	13	100
	**N1**	24	25	40	3	8	100
	**N2**	1	2	84	10	2	100
	**N3**	0	0	15	84	0	100
	**REM**	3	1	14	2	80	100

Notes: Shaded cells in the 5 × 5 matrix indicate the likelihood (%) that a PSG sleep state was correctly identified by Somfit. Non-shaded cells in the 5 × 5 matrix indicate the likelihood (%) of different types of misclassification errors by Somfit.

**Table 7:** Summary sleep variables (unfiltered subset, n = 26).

Variable	PSG M ± SD	Somfit (UF) M ± SD	Mean Bias M ± SD	Absolute Bias M ± SD	Paired *t*-Test t, df, *p*	Effect Size d, Low, High
Total sleep time (min)	455.4 ± 37.9	311.7 ± 156.6	−143.7 ± 155.0	152.0 ± 146.6	**−4.7, 25, <.001**	**−0.93, −1.38, −0.46**
N1 sleep (min)	31.2 ± 13.0	18.7 ± 11.3	−12.5 ± 13.0	13.9 ± 11.4	**−4.8, 25, <.001**	**−0.96, −1.41, −0.48**
N2 sleep (min)	229.8 ± 32.6	161.4 ± 91.9	−68.4 ± 87.0	80.3 ± 75.6	**−4.0, 25, <.001**	**−0.79, −1.22, −0.34**
N3 sleep (min)	94.0 ± 28.0	67.7 ± 48.0	−26.3 ± 46.9	42.4 ± 32.4	**−2.9, 25, .008**	**−0.56, −0.97, −0.14**
REM sleep (min)	100.4 ± 16.4	63.8 ± 46.1	−36.6 ± 43.5	43.4 ± 36.5	**−4.3, 25, <.001**	**−0.84, −1.28, −0.39**
Wake (min)	74.9 ± 36.8	64.1 ± 34.2	−10.8 ± 26.9	21.8 ± 18.8	−2.0, 25, .052	−0.40, −0.80, 0.00

Notes: UF = unfiltered; M = mean; SD = standard deviation; t = t-statistic; df = degrees of freedom; d = Cohen’s d; low, high = lower and upper bounds of the 95% confidence interval for Cohen’s d. Bolded values highlight results where *p* < .05 for the *t*-test and/or the 95% confidence interval for Cohen’s d does not include zero.

**Table 8:** Summary sleep variables (good-capture subset, n = 15).

Variable	PSG M ± SD	Somfit (GC) M ± SD	Mean Bias M ± SD	Absolute Bias M ± SD	Paired *t*-Test t, df, *p*	Effect Size d, Low, High
Total sleep time (min)	456.1 ± 41.3	431.0 ± 69.8	−25.2 ± 48.1	39.5 ± 36.4	−2.0, 14, .062	−0.52, −1.06, 0.03
N1 sleep (min)	27.7 ± 10.4	22.0 ± 9.9	−5.7 ± 9.5	8.1 ± 7.3	**−2.3, 14, .037**	**−0.60, −1.14, −0.04**
N2 sleep (min)	232.5 ± 36.4	225.3 ± 56.0	−7.1 ± 34.6	27.9 ± 20.5	−0.8, 14, .438	−0.21, −0.71, 0.31
N3 sleep (min)	92.3 ± 30.5	90.9 ± 48.2	−1.4 ± 39.1	29.4 ± 24.7	−0.1, 14, .894	−0.04, −0.54, 0.47
REM sleep (min)	103.7 ± 16.8	92.7 ± 34.8	−11.0 ± 31.3	22.7 ± 23.5	−1.4, 14, .194	−0.34, −0.85, 0.17
Wake (min)	75.8 ± 40.8	72.6 ± 39.2	−3.1 ± 18.6	14.1 ± 11.9	−0.6, 14, .524	−0.17, −0.68, 0.34

Notes: GC = good-capture; M = mean; SD = standard deviation; t = t-statistic; df = degrees of freedom; d = Cohen’s d; low, high = lower and upper bounds of the 95% confidence interval for Cohen’s d. Bolded values highlight results where *p* < .05 for the *t*-test and/or the 95% confidence interval for Cohen’s d does not include zero.

**Table 9:** Summary sleep variables (excellent-capture subset, n = 7).

Variable	PSG M ± SD	Somfit (EC) M ± SD	Mean Bias M ± SD	Absolute Bias M ± SD	Paired *t*-Test t, df, *p*	Effect Size d, Low, High
Total sleep time (min)	478.6 ± 16.7	489.1 ± 17.9	10.4 ± 14.8	14.1 ± 10.6	1.9, 6, .112	0.70, −0.16, 1.51
N1 sleep (min)	23.7 ± 9.6	18.9 ± 11.2	−4.8 ± 7.2	7.1 ± 4.5	−1.8, 6, .129	−0.67, −1.47, 0.18
N2 sleep (min)	246.9 ± 29.6	253.7 ± 43.0	6.8 ± 19.6	18.8 ± 4.9	0.9, 6, .394	0.35, −0.43, 1.10
N3 sleep (min)	95.3 ± 30.4	110.6 ± 53.9	15.4 ± 46.7	33.6 ± 33.7	0.9, 6, .417	0.33, −0.45, 1.08
REM sleep (min)	112.7 ± 17.4	105.8 ± 45.0	−6.9 ± 39.3	23.4 ± 31.0	−0.5, 6, .657	−0.18, −0.92, 0.58
Wake (min)	55.5 ± 17.0	50.9 ± 18.0	−4.6 ± 15.9	14.1 ± 6.8	−0.8, 6, .468	−0.29, −1.04, 0.48

Notes: EC = excellent-capture; M = mean; SD = standard deviation; t = t-statistic; df = degrees of freedom; d = Cohen’s d; low, high = lower and upper bounds of the 95% confidence interval for Cohen’s d.

## Data Availability

The data presented in this article are not readily available due to an agreement with the funder to protect the privacy of participants. Requests to access unidentifiable data should be directed to greg.roach@cqu.edu.au.
